# Recent Advances on Polymeric Beads or Hydrogels as Embolization Agents for Improved Transcatheter Arterial Chemoembolization (TACE)

**DOI:** 10.3389/fchem.2019.00408

**Published:** 2019-06-05

**Authors:** Yun-Ping Chen, Jiang-Ling Zhang, Yanhong Zou, Yun-Long Wu

**Affiliations:** ^1^Department of Oncology, The 910 Hospital of PLA, Quanzhou, China; ^2^Fujian Provincial Key Laboratory of Innovative Drug Target Research, School of Pharmaceutical Sciences, Xiamen University, Xiamen, China

**Keywords:** hydrogel, polymeric beads, drug delivery, TACE, cancer therapy

## Abstract

Transcatheter arterial chemoembolization (TACE), aiming to block the hepatic artery for inhibiting tumor blood supply, became a popular therapy for hepatocellular carcinoma (HCC) patients. Traditional TACE formulation of anticancer drug emulsion in ethiodized oil (i.e., Lipiodol^®^) and gelatin sponge (i.e., Gelfoam^®^) had drawbacks on patient tolerance and resulted in undesired systemic toxicity, which were both significantly improved by polymeric beads, microparticles, or hydrogels by taking advantage of the elegant design of biocompatible or biodegradable polymers, especially amphiphilic polymers or polymers with both hydrophilic and hydrophobic chains, which could self-assemble into proposed microspheres or hydrogels. In this review, we aimed to summarize recent advances on polymeric embolization beads or hydrogels as TACE agents, with emphasis on their material basis of polymer architectures, which are important but have not yet been comprehensively summarized.

## Introduction

Transcatheter arterial chemoembolization (TACE), which aimed to block the hepatic artery to inhibit the blood supply of solid tumor and to achieve localized chemotherapy, has now been popularly applied for liver cancer patients who were at a middle or late stage and were not suitable for surgical resection (Schwartz and Weintraub, 2008; Hyun et al., 2018). TACE was firstly proposed by Yamada et al. (1983), who showed that the interruption or reduction of hepatic artery blood supply during chemotherapy process was found to induce tumor necrosis or shrinkage without adverse reaction (Tsurusaki and Murakami, 2015). Since then, TACE has been widely applied in clinical practice, because this localized direct injection of chemotherapeutic drugs could lead to more than a 200 times local drug concentration increase and could achieve a fast curative effect, as well as a minimal side reaction (Varela et al., 2007).

The success of TACE was heavily reliant on the design of embolization reagents (Aliberti et al., 2017). Ideal embolization reagents should be able to (1) quickly and effectively block the blood supply upon intra-arterial injection; (2) release the embedded anticancer drugs for localized chemotherapy; (3) degrade after treatment for preventing thrombus; (4) potentially impair the angiogenesis. Initially, Lipiodol^®^ (ethiodized oil, in form of iodinated fatty acid esters of seed oil) was utilized to emulsify the anticancer drugs due to its lipophilic feature for intra-arterial chemotherapy, which was followed by Gelfoam^®^ (gelatin sponge) embolization and was recognized as conventional TACE. However, the manual fabrication of Gelfoam^®^,as well as its heterogeneous feature, led to the embolization effect of <3 days. Even worse, the injection time interval between Lipiodol^®^ and Gelfoam^®^ might lead to diffusion, systematic toxicity, and impaired patient tolerance of anticancer drugs, indicating the urgent need for better embolization agent designs.

**Figure 1:**
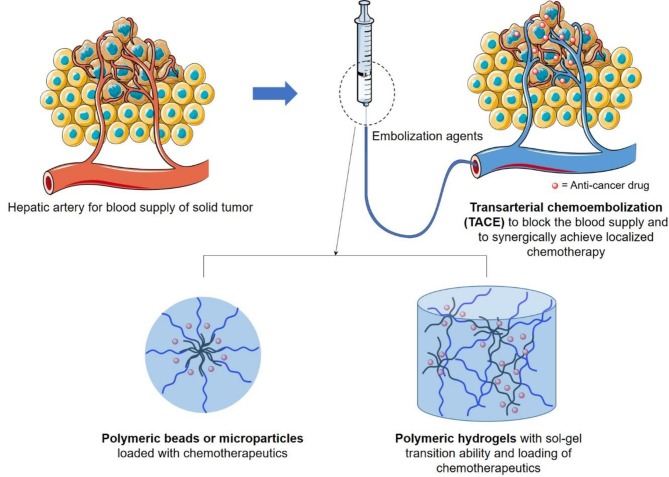
Schematic illustration of design of transarterial chemoembolization (TACE) agents, in terms of polymeric beads or hydrogels, to occulate the blood supply of liver tumor and to synergically achieve localized chemotherapy to reduce its side effects on surrounding healthy tissues.

Recently, polymers, especially amphiphilic polymers or polymers with both hydrophilic and hydrophobic chains which could self-assemble into microspheres, beads, or hydrogels (Hu et al., 2017a,b; Wu et al., 2017; Cai et al., 2018; Cheng et al., 2018b; Javanbakht and Namazi, 2018; Luo et al., 2018, 2019; Liu et al., 2019; Xu et al., 2019), have been successfully designed as a new generation of TACE embolization reagents, due to their ability to increase chemotherapeutic treatment efficiency and patient tolerance in comparison with traditional TACE by the formulation of Lipiodol^®^ and Gelfoam^®^ (Chen et al., 2016, 2018; Cai et al., 2017; Ding and Li, 2017; Fan et al., 2017a,b; Li and Loh, 2017; Li et al., 2017; Liu et al., 2017; Morimoto et al., 2017; Xiang et al., 2017; Yang et al., 2017; Zheng et al., 2017; Chan et al., 2018; Cheng et al., 2018a, 2019; Gao et al., 2018). In this report, we aim to summarize the current design of new generation embolization beads or hydrogels as illustrated in Figure 1, with emphasis on their material basis of polymer architectures, which are important but have not yet been comprehensively reviewed.

## Polymer Based Beads or Microparticles as TACE Reagents

Polymeric microspheres or beads with an ability to encapsulate chemotherapeutic reagents were developed as drug-eluting beads (DEB) and served as a significant advance. In comparison with traditional TACE formulation of Lipiodol^®^ and Gelfoam^®^, DEB with drug encapsulation in polymeric beads could effectively prevent the systematic diffusion of chemotherapeutics, especially at its first injection with high concentrations, and slowly release the embedded drug in a controllable manner to improve patience tolerance. As the first commercial DEB, DC Bead^®^ was fabricated by free radical polymerization of poly(vinyl alcohol) (PVA) with modification of N-acryloyl-aminoacetaldehyde (NAAADA), 2-acrylamido-2-methylpropane sulphonate sodium salt (AMPS), and cellulose acetate butyrate. DC Bead^®^ with sulfonate groups was able to encapsulate chemotherapeutic Doxorubicin (DOX), Irinotecan, Topotecan, or Epirubicin with H^+^ ions, by electron attraction. It was worth mentioning that a clinical study was conducted in 104 hepatocellular carcinoma (HCC) patients receiving treatments of DC Bead^®^ as DEB-TACE reagents by Bruix group (Burrel et al., 2012). The results showed that patients with DEB-TAC treatments could obviously receive a high dose of DOX without considering the undesired systematic circulation of injected drugs in comparison with conventional TACE formulation. This improvement might be beneficial for localized drug concentration increase and for overcoming drug resistance, indicating the great advantages on the safety of DEB-TACE. Furthermore, Gupta et al. (2011) demonstrated that chemotherapeutic DOX loaded superabsorbent microparticles could effectively increase the DOX intention concentration and increase the therapeutic effect in a liver tumor rabbit model. More importantly, Seki et al. (2011) explored the therapy procedure of 135 patients receiving TACE treatments with chemotherapeutic epirubicin-embedded superabsorbent polymer microparticles. The clinical results revealed that over 90% of patients receiving these treatments were not found to have hepatic artery damage and their 1- or 2- year survival rates were around 70 or 60%, respectively, indicating the practical value of drug eluting polymeric beads for patients with non-resected HCC.

Due to the non-bioresorbable nature of DC beads, many researchers have developed bioresorbable DEB by using a biocompatible and biodegradable polymer, to achieve long-term delivery of chemotherapeutics without considering the removal of the device after use. As a typical case, Golzarian's group designed a series of biodegradable DEB with a size of 300–700 μm by using chitosan and carboxylmethyl cellulose (Weng et al., 2011). Thanks to the carboxyl groups in these microspheres, DOX could be effectively loaded in DEB by electron interactions, with an efficiency of up to 0.3–0.7 mg DOX/sphere. Furthermore, this DEB exhibited a lysozyme dependent polymer degradation, which demonstrated a promising safe DOX carrier for transcatheter embolization application.

**Figure 2:**
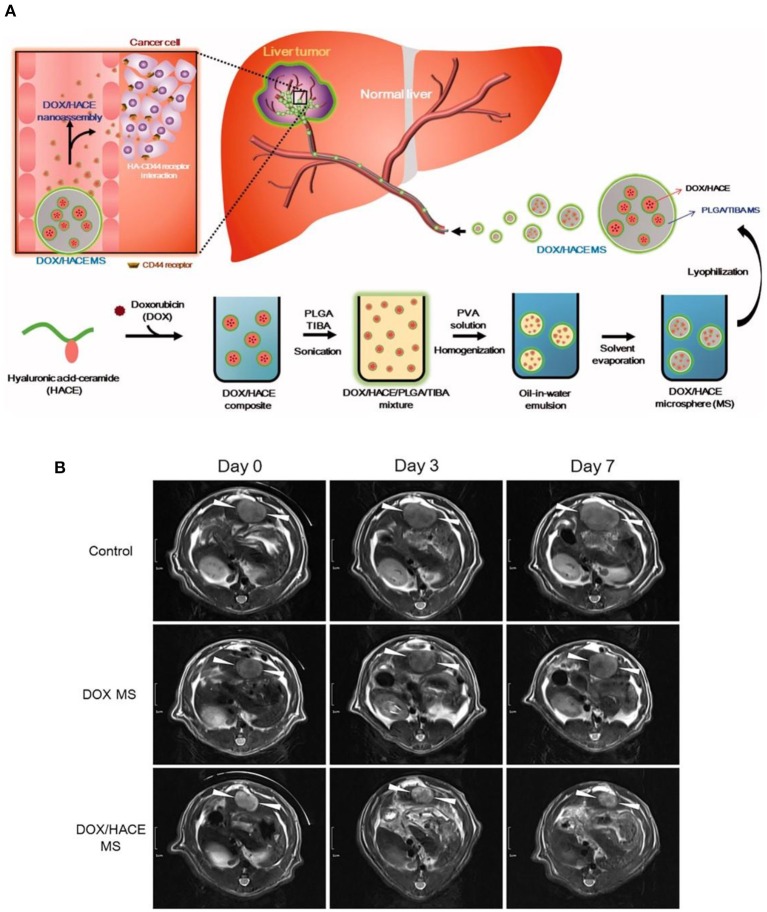
**(A)** Schematic illustration of tumor targeting HACE modified DOX/PLGA microsphere fabrication and treatment process as TACE reagent. **(B)** DOX/HACE MS (HACE modified DOX/PLGA microsphere) treatment could significantly decrease tumor size in McA-RH7777 tumor-implanted rat model, in comparison with control or DOX MS (DOX/PLGA microsphere) treatments. The tumor was recorded by magnetic resonance imaging. Scale bar is 1 cm. [Reproduced with permission from Lee et al. (2018), Copyright 2018 Taylor & Francis].

Recent endeavors also revealed that poly(lactic-*co*-glycolic acid) (PLGA), hydrophobic as well as biocompatible macromolecules, approved by the food and drug administration (FDA), could also serve as potential TACE reagents due to the high biodegradability. As a typical example, Choi et al. (2015) developed DOX embedded PLGA microspheres by the emulsion approach. Furthermore, they further trapped hyaluronic acid-ceramide (HACE) into the PLGA microspheres (MS) during the emulsion process (as shown in Figure 2A), to render TACE reagents with active liver cancer cell targeting ability (Lee et al., 2018). It was worth mentioning that intra-arterial injection of this tumor targeting DOX/HACE MS, with size of 13–44 μm, could significantly inhibit liver tumor growth in a McA-RH7777 liver cancer cell implanted rat tumor model, in comparison with the microspheres without targeting ability (as shown in Figure 2B).

It was worth mentioning that current commercial biodegradable microspheres still had their limitations. For example, Embocept^®^, a starch based microspheres with size of <100 μm, was only suitable for vessel embolization for a short time (<1 h) due to size limitation, which was not efficient for tumor necrosis (Yamasaki et al., 2012). Another case of Occlusin 500^®^, poly(lactide-*co*-glycolide) (PLGA)/collagen core/shell microspheres with size of 150–210 μm, required long degradation time (several months) with undesired inflammatory reactions (Owen et al., 2012). Ideal biodegradable microspheres should: (1) be able to induce vessel blockage for several hours to a few days, which is sufficient for tumor necrosis induction; (2) be able to degrade with non-harmful product and quick elimination; (3) be of uniform size ranging between 100 and 1,000 μm for board vessel embolization; (4) be easy in drug loading and be made of soft polymeric materials for micro-catheter injection (Hidaka et al., 2011). To achieve this design, Louguet et al. (2014) synthesized biodegradable polymeric microspheres with a size of 300–500 μm, which was made of amphiphilic poly(ethylene glycol) methacrylate (PEGMA) and biodegradable PEG-PLGA. These PEGMA/PEG-PLGA microspheres could be degraded in a few days with minimal toxicity effects induced by degradation products in fibroblast L929 cells and with a mild inflammatory reaction in an *in vivo* animal model, which showed promising application as safe biodegradable microspheres for TACE utilization.

## Amphiphilic Polymer as Cross-Linker of Microbeads

**Figure 3:**
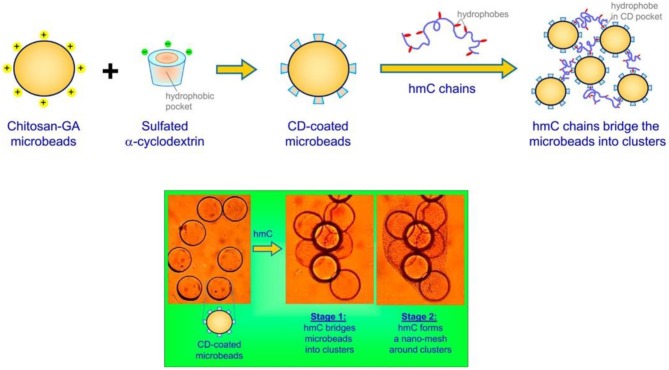
Schematic illustration of using amphiphilic hydrophobically modified chitosan (hmC) as cross-linking reagents for chitosan-glutaraldehyde (chitosan-GA) microbeads with surfaced modification of sulfated α-cyclodextrin (α-CD), as well as the self-assembly between hydrophobic chain of hmC and α-CD cavity, which could lead to formation of polymeric mesh outside microbeads for clotting applications. [Reproduced with permission from Arya et al. (2017), Copyright 2017 American Chemical Society].

To achieve a better embolization effect, material scientists tried to further conjugate microbeads into a polymeric mesh, using amphiphilic polymers. As a typical example, Arya et al. (2017) designed biopolymer chitosan-glutaraldehyde (chitosan-GA) microbeads with surface modification, i.e., having sulfated α-cyclodextrin (α-CD) as host molecules on the microbead surfaces, by taking the advantages of electronic attraction between cationic chitosan-GA microbeads and anionic sulfated α-CD (Figure 3, upper panel). Furthermore, the reaction between hydrophilic chitosan and hydrophobic n-dodecyl aldehyde rendered amphiphilic chitosan derivatives, whose hydrophobic segments could form an inclusion complex with α-CD due to its hydrophobic inner cavity nature. The experimental results showed that the addition of these amphiphilic chitosan derivatives with α-CD/chitosan-GA microbeads could lead to the bridging occurrence between microbeads and further formation of a polymeric mesh outside the microbeads (Figure 3, lower panel). Potentially, these amphiphilic macromolecule based microspheres with cluster formation ability could be utilized for reliable embolization and blood supply blockage.

## Composite Microbeads Made of Magnetic Nanoparticles and Polymer Matrix

It was also worth mentioning that the materials or polymeric basis of artery embolization agents were important, but it was not the only criteria for embolization design. For example, the size, homogeneity, and specific delivery of microbeads also served as key factors affecting the artery embolization efficiency. Typically, microspheres with size of 40–100 μm were suitable for hepatic artery end branch blockage; microspheres with a size of around 300 μm were utilized for tumor proximal vascular occlusion; while microspheres with size of 500–900 μm were applied for uterine arteries blockage for fibroid treatments; indicating that the size of embolization agents depended on the diameter of the target vessel (Maluccio et al., 2008). Furthermore, particles with undesired homogeneity might attribute to end organ damage or un-predictable distribution of microspheres, indicating the importance of controling the narrow size distribution or geometry of embolization agents (Stampfl et al., 2007). Last but not least, non-specific distribution of embolization agents after intra-arterial injection might induce undesired damage of healthy tissue (Hagit et al., 2010; Pouponneau et al., 2014).

**Figure 4:**
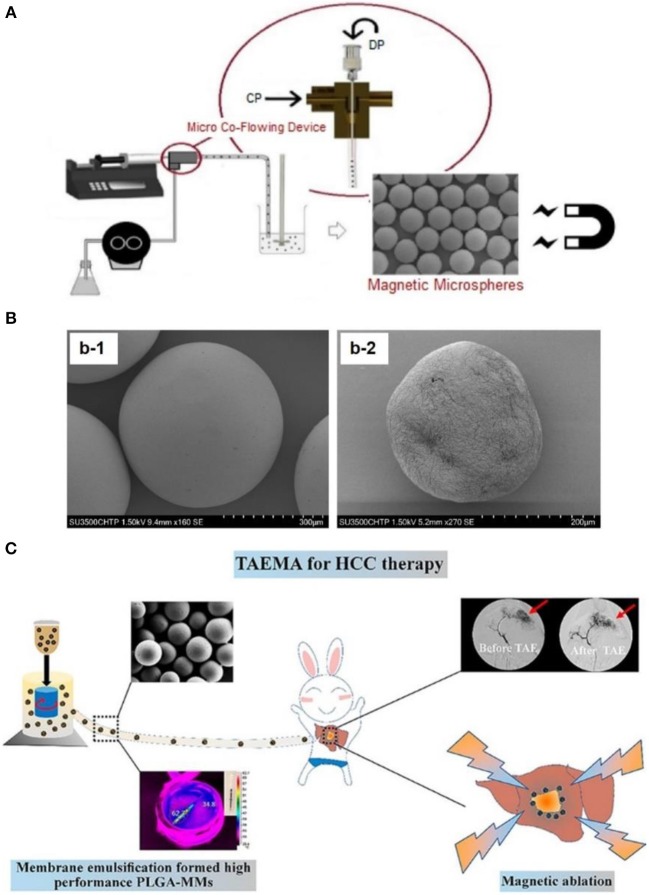
**(A)** Schematic illustration of fabricating magnetic microbeads by using micro-fluid technique, to fabricate magnetic nanoparticle embedded PLGA microparticles. **(B)** Scanning electron microscopy observation of magnetic microparticles embedding (b-1) 50 wt% magnetic iron oxide nanoparticles and (b-2) 60 wt% magnetic iron oxide nanoparticles. [Reproduced with permission from Nosrati et al. (2018), Copyright 2018 American Chemical Society]. **(C)** Fabrication of magnetic PLGA-MMs (PLGA-magnetic microspheres) by emulsion of PLGA polymer and iron oxide magnetic nanoparticles, which was utilized as liver arterial embolization agent in VX2 liver tumor of rabbit. PLGA-MMs could block the local blood supply and induce local temperature increase upon exposure to alternating magnetic field, which could cause synergetic magnetic ablation of tumor. [Reproduced with permission from Liang et al. (2017), Copyright 2017 American Chemical Society].

In order to overcome this problem, Nosrati et al. (2018) embedded magnetic iron oxide nanoparticles in a poly(lactic-*co*-glycolic acid) (PLGA) microsphere matrix using droplet microfluidics technology (as shown in Figure 4A), to fabricate homogeneous magnetic microbeads in a size range of 130–700 μm (as shown in Figure 4B). This gave potential to precisely monitor and control the accumulation of microbeads at disease vessel sites. Similarly, Liang et al. (2017) fabricated PLGA based magnetic microspheres (PLGA-MMs) by emulsion of PLGA polymer matrix and iron oxide nanoparticles. More interestingly, upon exposure to alternating magnetic field, these PLGA-MMs could not only act as embolization agents to block the blood supply in the VX2 liver tumor model of rabbit, but could also elevate the synergetic local temperature for magnetic ablation of liver tumors, as shown in Figure 4C. Last but not least, Hagit et al. (2010) synthesized composite microparticles using the copolymerization of hydrophobic 2-methacryloyloxyethyl (2,3,5-triiodobenzoate) (MAOETIB) and hydrophilic glycidyl methacrylate (GMA) as a core and a γ-Fe_2_O_3_ thin layer for shell coating, which were successfully explored as magnetic resonance imaging (MRI) as well as computed tomography (CT) contrast reagents to visualize the embolization procedure in a real-time pace. In short, composite microbeads, made of magnetic nanoparticles and polymeric matrix microspheres, demonstrated great advantages for targeted accumulation of embolization microbeads, thus might induce more desirable tumor ablation effects upon application of alternating magnetic field.

## Polymer Based Hydrogels as TACE Reagents

Due to the size limitation, microspheres or DEB were not suitable for large aneurysms, indicating the importance of developing more flexible embolic reagents. As a potential candidate, injectable polymeric hydrogel, with the ability to retain liquid status before injection but to form a solid hydrogel at the desired disease site, was favored and successfully applied to treat hemorrhage, cerebral aneurysms, or used as vessel sealants. For example, Golzarian's group had developed an *in situ* forming porous chemical crosslinking hydrogel made of carboxylmethyl chitosan and cellulose, with biocompatibility to endothelial cells, hemocompatibility, and bio-degradability in lysozyme solution (Weng et al., 2013). More importantly, the hydrogel precursor solution was injected, via a 5-F catheter, into an aneurysm sac and it successfully formed the solid hydrogel to fill the sac site and to prevent endoleakage. Further microcatheter injection of this hydrogel into rabbit kidney induced immediate renal artery occlusion and less injection volume in comparison with bead formulation (1–2 mL hydrogel vs. 5.5–7 mL microsphere), indicating the advantage of using hydrogels as embolization agents.

**Figure 5:**
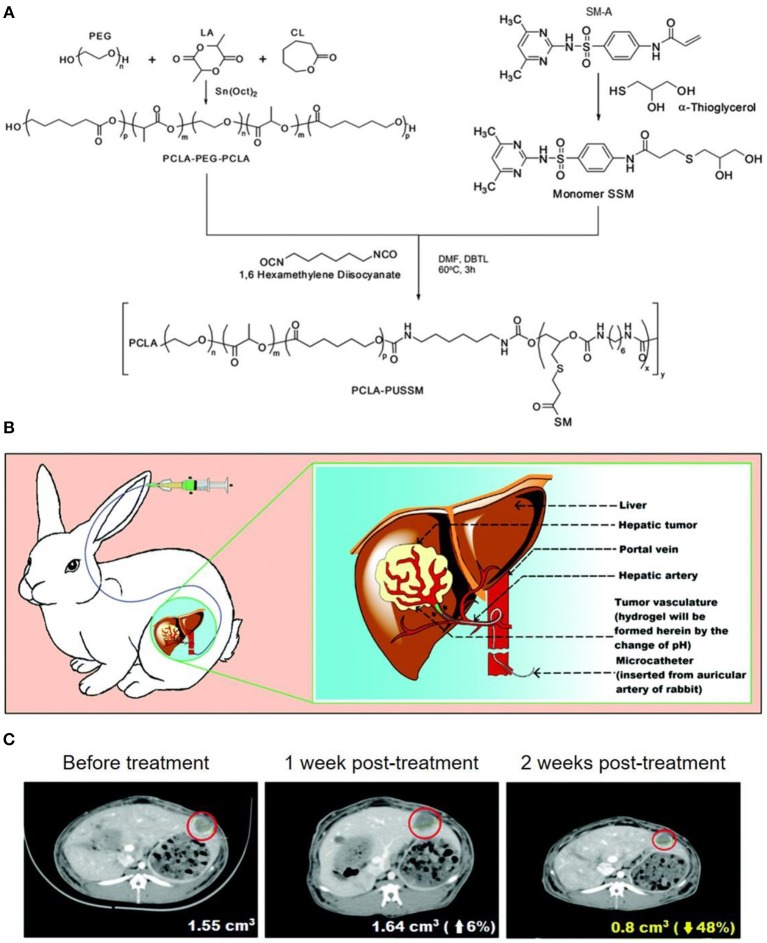
**(A)** Synthesis of amphiphilic anionic PCLA-PUSSM copolymer with pH-dependent hydrogel formation ability. **(B)** Illustration for the microcatheter mediated intraarterial injection of PCLA-PUSSM hydrogel in rabbit HCC model. **(C)** CT observations revealed the shrinkage of tumor size when using PCLA-PUSSM hydrogel as TACE agent. [Reproduced with permission from Nguyen et al. (2016), Copyright 2016 Royal Society of Chemistry].

Besides to chemical crosslinking hydrogel, physical crosslinking hydrogels with stimulus responsive phase changes were also popular design as TACE reagents. As a typical example, Nguyen et al. (2016) synthesized an amphiphilic anionic PCLA-PUSSM copolymer made of poly(ethylene glycol) (PEG), poly(ε-caprolactone-*co*-lactide) (PCLA), and poly(urethane sulfide sulfamethazine) (PUSSM), as illustrated in Figure 5A. The PCLA-PUSSM copolymer solution remained liquid status at pH 8.5 and experienced fast phase change and solid status hydrogel formation upon pH decrease. By taking this unique pH responsive phase change process, this PCL-PUSSM hydrogel could be intra-arterially injected into hepatic tumor of VX2 rabbit model, and achieved embolization as well as controllable release of embedded DOX in a sustainable manner, as shown in Figure 5B. Animal model evaluation revealed that this PCLA-PUSSM hydrogel could effectively perform the chemoembolization effect and release DOX in a sustained manner to inhibit the tumor growth, as shown in Figure 5C.

Similarly, Lym et al. (2016) designed a pH-responsive PCL-PEG-SM copolymer by free radical polymerization of PEG, poly(ε-caprolactone) (PCL), and sulfamethazine (SM). More interestingly, the aqueous solution of PCL-PEG-SM copolymer experienced a sol-to-gel transition from pH 8.0 to pH 7.4 at 37°C, and could achieve a sustained release of DOX for up to 4 weeks. It was also worth mentioning that the embolic formulation of PCL-PEG-SM copolymer solution at pH 8.0 could be intra-arterially administrated in rabbit VX2 liver tumor model.

In short, injectable hydrogel with environment responsive ability had been successfully applied in TACE procedure for hepatocellular cancer (HCC) treatment in an animal model, and it exhibited the ability to stably maintain high drug concentration at the tumor site. Hence, the design of biocompatible and biodegradable polymeric hydrogels might serve as a practical TACE agents, which could be further combined with a wide spectrum of chemotherapeutics or X-ray contrast agents to show improved performance compared to traditional Lipiodol^®^ or Gelfoam^®^ formulation.

## Conclusions and Perspectives

In conclusion, this review showed the recent progress of polymeric TACE agents, in terms of polymeric beads or microparticles, polymeric meshes by crosslinking beads, polymeric hydrogels, with great potential for treating patients with unresectable HCC. More importantly, the design of these polymeric TAC agents, especially polymer backbone materials, degradability, size, or geometry, was important for safe and efficient tumor blood supply blockage, as well as localized and sustained release of chemotherapeutics to achieve better liver cancer therapy.

**Figure 6:**
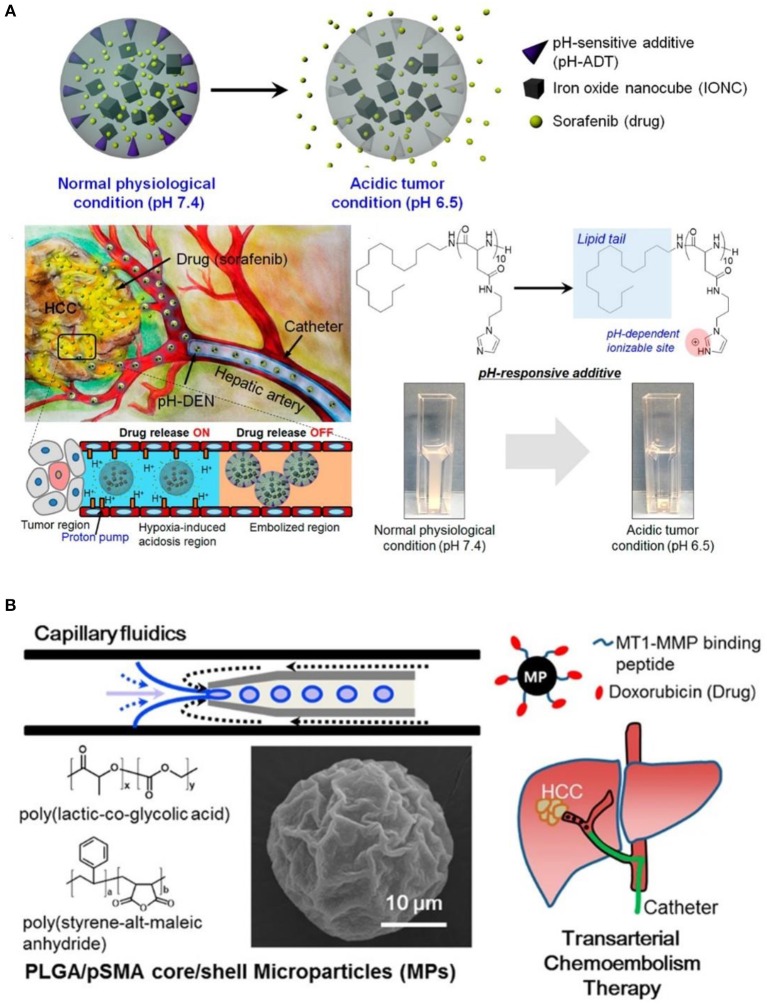
**(A)** Illustration of environment sensitive drug-eluting composite particles, as well as their low pH responsive chemotherapeutics release in hepatic vessels. [Reproduced with permission from Park et al. (2016), Copyright 2016 American Chemical Society]. **(B)** Synthesis of tumor targeting PLGA/pSMA microspheres with conjugation of MT1-MMP binding peptide and chemotherapeutic doxorubicin, for more precise transarterial chemoembolism therapy. [Reproduced with permission from Davaa et al. (2017), Copyright 2017 American Chemical Society].

It was also worth mentioning that, in addition to the above mentioned formulations of microbeads or microspheres, cross-linked microspheres, and hydrogels, Lipiodol oil embedded PEO-PPO-PEO/PEG (poly(ethylene oxide)-poly(propylene oxide)-poly(ethylene oxide)/poly(ethylene glycol) composite capsules containing paclitaxel (PTX) (Bae et al., 2007), poly(ethylene glycol) PEG liposomes containing 5-fluorouracil (5-FU) (Pohlen et al., 2011), methoxy-poly (ethylene glycol)-*block*-poly(ε-caprolactone) (mPEG-*b*-PCL) micelles carrying doxorubicin (DOX), as well as radio-active rhenium-188 for combined radiotherapy and chemotherapy, were also actively applied for hepatic artery embolization and cancer therapy (Shih et al., 2015). This demonstrates the wide applications of polymeric materials for TACE applications. Furthermore, besides to polymeric embolization agents, injectable precursor of polar lipid phytantriol at cubic liquid crystalline phase (Han et al., 2010), and drug eluting composite containing pH sensitive lipid-peptides conjugation (octadecylamine–poly (API-_L_-Asp)_10_) as shown in Figure 6A (Park et al., 2016), could also be designed for vascular embolization, and to achieve localized drug sustained release. This could also be further combined with polymeric embolization agents for enhanced therapeutic effect. Last but not least, functional peptide could also be conjugated to polymeric microparticles for targeted delivery of embolization beads. As a typical example, Davaa et al. (2017) linked matrix metalloproteinase responsive peptide (MT1-MMP binding peptide) to poly(lactic-*co*-glycolic acid/poly-(styrene-alt-maleic anhydride) (PLGA/pSMA) microspheres, which exhibited enhanced accumulation in MT1-MMP overexpressing Hep3B cells, as shown in Figure 6B. More interestingly, these peptide conjugated microspheres could accumulate in hepatic vessels for up to 24 h without undesired diffusion to lung, and they significantly inhibited the tumor growth in a Hep3B xenografted mice model. Hence, we can expect great potential for the design of functional polymeric materials with nationally designed stimulus-responsive crosslinking segments or biodegradable groups, for embolizing tumor blood supply in a fast and controllable manner, as well as ensuring biodegradability in order to be safely removed after use. This might significantly broaden the choice of TACE agents for more successful and precise HCC therapy.

## Author Contributions

Y-LW and Y-PC initiated the project. Y-PC, J-LZ, YZ, and Y-LW searched the data base, wrote, and finalized the manuscript.

### Conflict of Interest Statement

The authors declare that the research was conducted in the absence of any commercial or financial relationships that could be construed as a potential conflict of interest.
